# Experiences of rehabilitation in young elite athletes: an interview study

**DOI:** 10.1136/bmjsem-2023-001716

**Published:** 2023-11-03

**Authors:** Linda Ekenros, Cecilia Fridén, Philip von Rosen

**Affiliations:** 1 Department of Neurobiology, Care Sciences, and Society (NVS), Division of Physiotherapy, Karolinska Institutet, Stockholm, Sweden; 2 Department of Women's and Children's Health, Karolinska Institutet, Stockholm, Sweden; 3 School of Health, Care and Social Welfare, Physiotherapy, Mälardalen University, Mälardalen, Västerås, Sweden

**Keywords:** Adolescent, Injury, Muscle damage/injuries, Qualitative Research

## Abstract

Even though injuries are common in elite youth sports, rehabilitation experiences are limited explored in young athletes. This study explored rehabilitation experiences in athletes with a previous injury studying at sports high schools. Twenty-six (14/12 females/males) young elite athletes (age 15–19 years) from 11 individual/team sports were interviewed in focus groups about the rehabilitation experiences following a sports injury. Data were analysed using content analysis. The results led to four main categories identified: ‘High-quality rehabilitation’, ‘Lack of communication between healthcare and coach’, ‘Various consequences of injury’ and ‘No clear path to accessing rehabilitation’. The athletes valued that the healthcare providers had high expertise, were clear and signalled secureness and confidence. It was also important to receive help with rehabilitation as rapidly as possible. The athletes perceived that they felt responsible for providing information regarding rehabilitation progression between healthcare providers and their coach. It was also challenging not to be able to participate in training and competition while injured — causing a sense of frustration and fear of falling behind their peer athletes in terms of development and performance. The athletes described that no well-defined medical teams at the sports high schools were available— instead medical help was offered in several other ways. Based on our findings, the collaboration and communication between the healthcare providers and coaches need to be improved, addressing the rehabilitation content, progress and access to rehabilitation. Through these actions, the rehabilitation process will be more adjusted to the needs of young elite athletes.

**WHAT IS ALREADY KNOWN ON THIS TOPIC**

- Current rehabilitation practice might not align with the needs of young elite athletes.

**WHAT THIS STUDY ADDS**

- Insight into rehabilitation and injury experiences are provided—exploring the specific needs of the young elite athlete.

**HOW THIS STUDY MIGHT AFFECT RESEARCH, PRACTICE OR POLICY**

- By developing and improving collaboration between coaches and healthcare providers around rehabilitation accessibility, content and progress, the specific needs of the young elite athlete are recognised.

## Introduction

At national sports high schools in Sweden, adolescent athletes can practice their sport to enhance athletic performance while completing a high school degree. However, high exposure to sports has been associated with an increased risk for musculoskeletal injuries in youth athletes.^1 2^ In young elite athletes, the average weekly prevalence of substantial injury is 17%.^3^ Besides causing physical harm, injuries also affect mental health.^4 5^ Therefore, healthcare providers, such as physiotherapists, physicians, sports psychologists, nutritionists, and so, have an important role in meeting the various needs of youth athletes.

Rehabilitation in sports medicine normally focuses on restoring function and performance to preinjury, safe return to sports and reduction in the risk of reinjury.^6^ Following injury, youth athletes normally want to return to a preinjury level of fitness and performance as rapidly as possible. Since youth athletes have no or little experience of being injured, it is understandable that the injury period is perceived as stressful. In college athletes, the onset of injury has been associated with experiences of negative thoughts and depressed mood.^7^ In adult athletes, an injury could lead to feelings such as frustration, anxiety, anger and upsetness.^8^ Diminished self-identity while recovering from injury has also been found in adult and youth athletes.^5 9 10^

Seeking social support from family and friends seems to be a common strategy to handle injury emotions. Social support is associated with coping and enhanced rehabilitation following a sports injury.^11^ However, young athletes normally move from home when admitted to sports high schools, resulting in changes in social support. Therefore, healthcare providers may need to provide additional support to the athlete through the rehabilitation stages, as this has been shown to influence rehabilitation and well-being positively.^12 13^

So far, few studies have addressed the challenges of rehabilitation in young elite athletes. Examples of challenges identified are related to coping with reduced sports activity, feelings of exclusion, fear of movement and challenges when returning to sports.^5^ These consequences create stressful and possibly new and adverse situations for a young athlete, which could lead to psychosocial disturbances such as depressed mood or depression.^14 15^ Therefore, it is important that athletes enter rehabilitation at an early stage, which may affect their return to sports.^7^ As coaches are key figures in the sporting environment, they also play an important role in return to sports decisions.^16^ Therefore, there is a need for close collaboration and open communication between the coach and healthcare providers. However, there are limited data on how young elite athletes perceive this collaboration.

Exploring experiences of rehabilitation in young elite athletes is relevant and important for all stakeholders involved in rehabilitation. For instance, it can lead to barriers and facilitators of rehabilitation being identified. Thereby, the rehabilitation process can be adjusted to the needs of young elite athletes. So far, a limited number of studies have targeted rehabilitation experiences in young elite athletes, especially those studying high school sports. Therefore, this study explored rehabilitation experiences in young elite athletes with a previous injury studying at sports high schools.

## Methods

This study used a phenomenological qualitative design and data were analysed with an inductive content analysis approach,^17^ involving adolescent elite athletes from individual and team sports studying at sports high schools.

### Participants and recruitment process

Coaches at sports high schools were contacted by email regarding recruiting athletes to this study. A total of 15 coaches from different sports were approached, of which 10 accepted the invitation to participate in an interview-based study. The athletes who met the inclusion criteria were asked by their coaches if they were interested in participating in an interview-based study. To be included in this study, the athletes needed to attend a sports high school and have a history of being injured, which affected participation in their main sport for at least four continuous weeks within the last year. This resulted in 32 athletes (aged 15–19 years) being contacted by the authors. To maximise experiences and insights about rehabilitation experiences, athletes with different injury types, sexes, sports and from different parts of Sweden were approached. In total, 26 athletes (14/12 females/males) were enrolled (table 1). The athletes reported to have had acute injuries (eg, ankle sprain, fractures, traumatic brain injury, anterior cruciate ligament injury) and overuse injuries (eg, stress fracture, patellar tendinopathy, patellofemoral pain syndrome), of which rehabilitation lasted between 2 and 12 months.

**Table 1:** Participants’ characteristics by focus group

Group	Females/males	Sports	Injury	Length of rehabilitation
Focus group 1	3/2	Sailing, swimming	Subacromial pain, back pain, overuse injury elbow	4–8 months
Focus group 2	2/3	Basketball	Ankle fracture, ankle sprain, patellar tendinopathy, overuse injury knee	3–10 months
Focus group 3	2/1	Bandy, orienteering	Ankle sprain, medial tibial stress syndrome, patellar tendinopathy, patellofemoral pain syndrome	3–12 months
Focus group 4	3/1	Cycling, mountainlike	Patellofemoral pain syndrome, stress fracture hip, back pain	2–8 months
Focus group 5	1/2	Cross-country skiing, football	Back pain, overuse injury shoulder, anterior cruciate ligament injury	4–12 months
Focus group 6	2/1	Cross-country skiing, triathlon	Back pain, patellofemoral pain syndrome, overuse injury knee	2–6 months
Focus group 7	1/2	Football, orienteering	ankle fracture, knee collateral ligament injury, patellofemoral pain syndrome	3–10 months

### Context of participants

Young elite athletes are typically between 15 and 19 years of age when they start studying at a sports high school. The education lasts for 3 or 4 years; at the school, the athletes can combine elite sports and a high school education. Sports coaches and teachers are in charge of the sports education at the schools. The long-term goal of education is to help athletes reach the international elite level in their specific sport. Most students leave home when admitted to sports high school and start living alone or with other athletes close to the schools. This may inadvertently lead to changes in social support responsibility and increased pressure on oneself as competitiveness increases. The schools often have established collaboration with medical personnel regarding injury rehabilitation; a few schools even have employed medical personnel responsible for the athletes’ rehabilitation.

### Interview guide

Two experienced physiotherapists in sports medicine initially developed a semistructured interview guide. The interview guide consisted of questions addressing the experience of being injured, injury rehabilitation and support from healthcare providers (online supplemental file 1). Two pilot interviews were conducted with non-elite youth athletes in focus groups (three athletes in each group). After these interviews, some minor changes to the wording were made to the interview guide. The pilot interviews were not used in the data analysis.

10.1136/bmjsem-2023-001716.supp1 Supplementary data

### The focus group interviews

The recruited athletes formed seven focus groups (3–5 per group), mixing male and female athletes from different sports. Focus group interviews were chosen since this is an appropriate method for sharing perspectives and discussing experiences.^18^ Due to logistic reasons, two of the interviews involved athletes from only basketball and cycling, respectively, while the remaining interviews involved athletes from at least two sports.

### Data collection

The interviews were carried out between 2019 and 2021. All interviews were planned to be performed within a season. However, the recruitment of participants was delayed due to COVID-19. The interviews were performed by a physiotherapist with clinical experience in sports medicine (PvR, PhD) and two physiotherapist students in the final semester. The physiotherapist with clinical experience in sports medicine had previous experience conducting qualitative research interviews. The physiotherapist students had not conducted interviews in qualitative research. The semistructured interview guide was discussed among the interviewers to ensure consistency across the interviews.

At the time of the interviews, written informed consent was obtained from the athletes regarding participation in the study. The interviews lasted between 40 and 65 min (an average of 55 min) and were all digitally audio recorded. The interviews were then transcribed verbatim (56 pages).

### Data analysis

An inductive content analysis approach, as described by Graneheim and Lundman,^17^ was used to analyse the interview data. Following Graneheim and Lundman,^17^ the analysis process started with data familiarisation, generation of initial codes and then categorisation of the data by searching for themes. The text material was initially read thoroughly several times. After that, the text was coded into meaning units, that is, words and statements relevant to the aim of the study. All meaning units were then condensed and grouped into subcategories and categories. It was ensured that the categories were internally homogenous and externally heterogeneous. The analysis was carried out by one of the authors (PvR) and was then discussed among the authors. The analysis process was iterative, with an ongoing back-and-forth process between the data and its various parts. Finally, all categories were reviewed, defined and named. The athletes did not provide any feedback on the results of the data analysis. An example of the analysis process is presented in table 2.

**Table 2:** Example of the data analysis process

Meaning unit	Condensed meaning unit	Code	Subcategory	Main category
I think it is very important that the physiotherapist should be confident and know what he is talking about.	A physiotherapist should be confident and know what he is talking about.	Be confident and have high expertise	Role of healthcare provider	High-quality rehabilitation
If you know the steps in rehabilitation…it is much easier. You know what to do and when to be back on track. It reduces my stress.	Knowing the steps in rehabilitation leads to reduced stress	Steps in rehabilitation are known	Rehabilitation plan	
I don’t like being referred to as the injured person by my physiotherapist*…*I am an athlete. That is how I described myself. (New person) I agree with you. We just happened to have injuries…I am still a basketball player.	Wants to be perceived as an athlete, not as an injured athlete	Athlete identification	How to be perceived	

## Results

**Figure 1:**
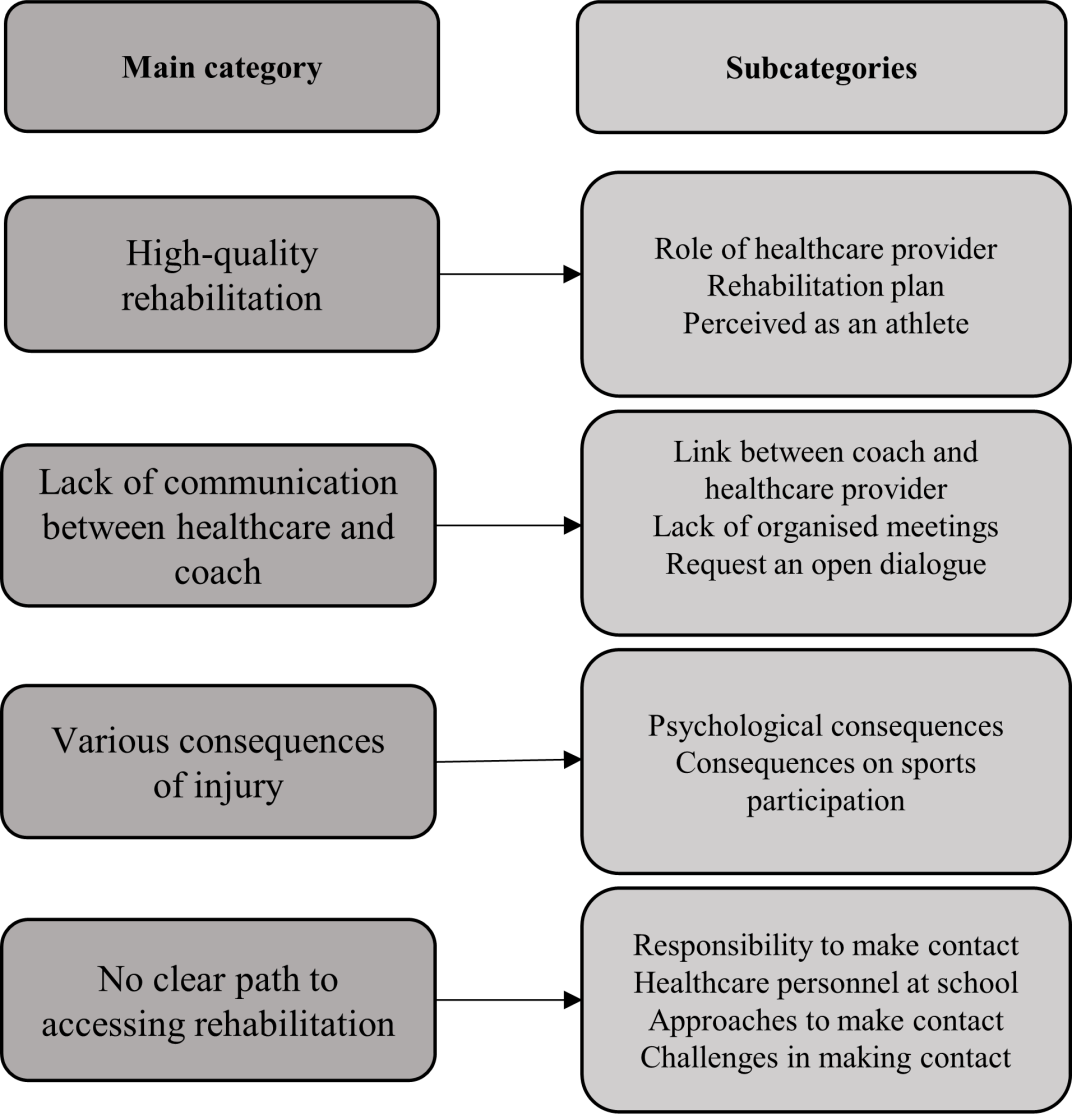
Results based on the manifest qualitative content analysis.

The inductive content analysis led to 12 subcategories and 4 main categories being identified. These main categories were ‘High-quality rehabilitation’, ‘Lack of communication between healthcare and coach’, ‘Various consequences of injury’ and ‘No clear path to accessing rehabilitation’ (figure 1).

### High-quality rehabilitation

The athletes valued that the healthcare providers had high expertise, were clear, and signalled secureness and confidence. It was also important to receive help with rehabilitation as rapidly as possible. The athletes also described the importance of having someone to share concerns and injury experiences with.

And it’s helped a lot (talking to a physiotherapist) because I was kind of rotting inside until I kind of started talking to her…it was like one of the best things…during my time being sick or injured. (Interview 6)

The athletes valued having a clear and structured rehabilitation plan. This was calming and reduced the pressure, as for many athletes, participating in rehabilitation was a new process.

So it was very clear in that way, that you kind of start from scratch, and there was a clear plan. She was the one who told me that you should do this many times a day, this many reps, and it was very nice and secure. (Interview 4)

If their physiotherapist was responsible for many athletes, it was difficult to get enough time, feedback and time for follow-up. The athletes then described feeling unseen and ignored.

If you are doing rehabilitation, it is important that your physiotherapist watches you and tells you what to do. Otherwise, it is lonely, and you don’t know the next step or right way to exercise*.* (Interview 1)

The athletes requested to be treated as ‘athletes’ while injured and not as ‘injured athletes’.

I am not an injury…and don’t want to be named a “person with injury”. I am a cyclist. (Interview 5)

### Lack of communication between healthcare and coach

The athletes perceived that they felt responsible for providing information regarding rehabilitation progression between healthcare providers and their coach. This information concerned rehabilitation content, progress and integrating rehabilitation with normal practice. Most athletes considered this difficult as sometimes the athlete did not understand the physiotherapist fully and was not sure what the next step in rehabilitation was. They also felt pressure to provide the correct information. Most athletes described no communication between their coach and healthcare providers.

My coach and my physiotherapist have no contact at all. All communication is through me…I explain what the physiotherapist has said. (Interview 3)

The athletes requested mutual meetings with their healthcare providers and coaches. They also wanted an open dialogue and that all rehabilitation steps were discussed. A few athletes also suggested that a mutual monitoring system could be used to share information about rehabilitation. Most athletes requested that their physiotherapist visit their practice and adjust parts of the training practice, if necessary while reducing the gap between the coach and healthcare providers.

When you’re alone and doing rehabilitation, you don’t think about what you’re doing wrong, if you do something wrong? I just think you just want to get back as quickly as possible. Once you do the rehabilitation, someone is there and checks and acknowledges if you did something wrong. If you had a physiotherapist there to help you and talk to the coach, I think that would do a lot. (Interview 4)

Most athletes described pressure to decide when they should return to sports. Since, most often, no communication occurred between their coach and physiotherapist, the athlete felt responsible for the decision and did not know whether they should participate fully, partially, or not at all.

That’s quite difficult to announce that…I can’t train that much today. It would have been easier if it was a physiotherapist who said that she can’t train this much…because then… it is someone else who has decided it, not me…You are afraid… that you will get the reactions that, shouldn’t she be here, why not? You can’t be that injured… (Interview 5)

The coach and physiotherapist, it’s not like they usually talk a lot, but it feels like you…and I think that’s kind of what’s hard too, that you have to announce yourself that no, I can’t train that much today. It would have been easier if it was a physiotherapist who said no, she can’t exercise this much. Because then it turns out that it is someone else who has decided it, not me. That particular communication is not very good, I think. (Interview 1)

### Various consequences of injury

The athletes described that it was challenging not to be able to participate in training and competition while injured. Watching other athletes participate created a feeling of frustration and fear of falling behind their peer athletes in terms of development and performance. When the athletes were injured, they felt that they did not belong to the rest of the group and described that the injury affected their social life and led to isolation.

You start to feel lonely and miss your friends that you normally see every day. Suddenly, you have to do everything by yourself*.* (Interview 2)

So it has been very tough to see many people sailing and being able to improve when I can’t. (Interview 5)

Most athletes feared that returning to play could make the injury worse. Yet, several athletes had concerns regarding how they would be noticed if they did not participate in sports. The athletes also described a sense of loneliness during rehabilitation because they often had to perform it alone.

Every time you go to rehab… you’re in the gym by yourself, and you sit there with your paper with exercises, you do rehab day in and day out… you lose motivation. If you had a physiotherapist there to help you… I think it would make the whole process much easier. (Interview 3)

### No clear path to accessing rehabilitation

The athletes described that no well-defined medical teams were available at the sports high schools. Instead, the athletes described that medical help was offered in a number of other ways. A few athletes had access to a physiotherapist who worked partially at the school, and some athletes chose to travel to their hometown to access a physiotherapist. Some athletes could chat with healthcare providers through a mobile application (app).

We recently have started to use an app that can give you advice and direct you to help with your injury (online). It works okay, but I think it is more important to meet someone (in real life) who can tell you what to do to get rid of the injury. (Interview 3)

All athletes were responsible for making contact with the healthcare providers, and the coach was not involved in the booking process.

We have no physiotherapist at the school. You therefore need to find and directly make contact, which is difficult as I am not used to finding the right help for my knee (injury). (Interview 6)

Many athletes described access to rehabilitation as complicated and requested a simpler way to enter rehabilitation.

It would be a bit more practical to have access through school to avoid the costs…but also just easier to have access and get in touch with someone (in rehabilitation). (Interview 2)

If the relationship with the healthcare provider did not work or the rehabilitation was not progressing, the athlete was responsible to contact healthcare personnel again. This was perceived as challenging, and the athletes requested more guidance to find the right healthcare provider.

For me…it is difficult to know to whom I should turn. I have no experience of getting help for my injuries and rehabilitation*.* (Interview 1)

## Discussion

Exploring young athletes’ experience of rehabilitation highlighted four main categories; aspects that the athletes value in rehabilitation, lack of communication between coach and healthcare provider, injury consequences and different ways to access rehabilitation. The results are discussed, targeting rehabilitation actions and injury prevention strategies.

To our knowledge, few studies have targeted which aspects young elite athletes value in terms of rehabilitation. Instead, high-quality rehabilitation is almost always described based on a specific injury in sports medicine,^19–21^ while a youth athlete perspective is not known. Based on our findings, the athletes valued that the healthcare provider had high expertise and used clear and open communication. Good communication skills have previously been described as vital by adult athletes.^22 23^ In addition, the athletes valued rapid help to enter rehabilitation and having a rehabilitation plan. We could also demonstrate that the athletes wanted to be treated as athletes, not as ‘the one-with-an-injury’. In line with studies on adult and youth athletes, injury has been found to threaten athletes’ self-identity,^5 9 10^ and our findings support this assumption.

The athletes also valued the ability to share injury experiences with their healthcare providers. An open dialogue with the athlete, initiated by the healthcare provider, has previously been highlighted as important. This is particularly important as many injured young athletes may not share their feelings and concerns without prompting.^24^ The athletes also wanted their physiotherapists to provide mental support during rehabilitation. Mental support has been suggested as an integral part of all sport-injury rehabilitation,^22^ likely enhancing the athletes’ motivation for rehabilitation and counter their feelings of isolation. Group rehabilitation sessions, involving multiple athletes that share injury experiences and feelings, might also be one approach to countering the feeling of isolation.

Our results also demonstrate that the athletes were responsible for communicating between their coach and healthcare provider. Clear and open communication between athletes, family, coaches and healthcare providers is essential for a good return to sports decision,^25^ and individualised care requires clear communication pathways. Based on our findings, this was not the case for the interviewed young elite athletes. Instead, the athletes had to relay information about their rehabilitation between the coach and healthcare provider. By letting the young athlete act as the messenger, miscommunication will likely occur, increasing the risk of reinjury. This has been shown in adult athletes.^23^ We, therefore, recommend that healthcare providers take time to inform the athlete’s coach about rehabilitation content and progress while avoiding using the young elite athlete as the primary messenger. However, the athlete needs to consent that personal information is shared with the coach. If not, the patient’s privacy may be at risk; effective communication between athlete, coach and healthcare provider is vital for shared decision-making about return to play decisions and optimising the rehabilitation.^26^

In the integrated model of psychological response to sport injury by Wiese-Bjornstal *et al,*^27^ it is suggested that personal (eg, history of injury) and situational factors (eg, social support, coach influence) affect the individual’s cognitive appraisal, that is, how athletes respond and react to an injury. In this study, the athletes described several injury consequences, for example, psychological and behavioural consequences, consistent with responses described in the integrated model.^27^ For instance, the athletes felt excluded while injured, demonstrated in previous research.^5 28^. They also described a sense of loneliness during rehabilitation, highlighting the importance of social support during all rehabilitation phases. The athletes also suggested that the injured athlete participate in training practice as much as possible and that their physiotherapist could join the practice to monitor and optimise the rehabilitation, which may reduce the feeling of exclusion. The athletes also described concerns and felt pressured not to participate in sports. This was often decided by the healthcare provider or the coach that the athlete should not participate in their sport. The athletes also described fears and pressure to return to sports, which had previously been highlighted in adult athletes.^11^ Fear of reinjury and low confidence have been described as barriers to returning to sports.^29^ According to a consensus statement, return to sports should ideally be decided by healthcare providers, athletes and coaches collaboratively,^30^ and not as in this study where some athletes felt responsible for taking this decision without support.

There was no easy way to access rehabilitation for the young elite athletes, and the athletes described high levels of responsibility in finding help for their injuries. Even if an injury is associated with negative responses,^7 8^ navigating to finding the right type of care has been perceived as an additional stressor in young athletes.^5^ Developing multidisciplinary healthcare teams specialised in helping young athletes may enhance rehabilitation and facilitate access to rehabilitation. Today, most sports high schools do not have employed medical personnel. Therefore, coaches should support young elite athletes in accessing rehabilitation to bridge the gap in accessing rehabilitation.

The results of this study should be viewed in light of its methodological limitations. Due to the study design, the findings cannot be generalised to the population of all injured athletes. However, the interviewed athletes represented different sports and schools, which enriches the perspective of athletes’ experiences and enhances credibility. Conducting several interviews may have led to the interviewers getting more insight into the phenomenon, possibly affecting the follow-up questions and dependability.^17^ The interviews were performed by three different interviewers which may influence the follow-up questions. However, the semistructured interview guide was used in all interviews. The duration of the rehabilitation varied greatly across athletes, which could be seen as a limitation. The data were collected over several semesters since injury varied across athletes, affecting how the rehabilitation was recalled. However, conducting the interviews in focus groups likely promoted a diversity of experiences. Still, the disadvantage of focus group interviews is the tendency for participants to dominate or influence the other participants, which may have influenced our results. It remains possible that data saturation was not achieved, particularly as fewer athletes from team sports, compared with individual sports, were included. Trustworthiness was ensured using several criteria, such as applying a sample strategy (maximum variation) in the recruitment of athletes, using pilot interviews to become more familiar with the interview process and a clear description of the steps of data collection and analysis. Further research should target the coach and healthcare providers’ experiences from working with injured adolescent elite athletes and thoughts on how rehabilitation can be improved.

## Conclusions

Our findings emphasize the importance of adjusting the rehabilitation process to young elite athletes and the context of sports high school. This could partly be achieved by improving the collaboration and communication between the healthcare providers and coaches. It also seems important that healthcare providers inform the athlete’s coach about rehabilitation content and progress and that coaches support young elite athletes in accessing rehabilitation. Through these actions, some of the needs and concerns of young elite athletes with rehabilitation are addressed.

## Data Availability

Data are available upon reasonable request. The data that support the findings of this study are available on reasonable request from the corresponding author.
