# Supplementary material for: Experiences of rehabilitation in young elite athletes: an interview study
Source: BMJ Open Sport Exerc Med. 2023 Nov 3;9(4):e001716. doi: 10.1136/bmjsem-2023-001716 (PMC10626772; doi:10.1136/bmjsem-2023-001716)
Supplement: Supplementary data [file bmjsem-2023-001716supp001.pdf]

Interview guide

1. Can you describe what a typical training week looks like?

2. Can you tell us about an injury you have suffered during high school? How and where did it happen?

3. Can you describe how the injury was handled?

4. Can you tell us about how the rehabilitation of the injury went?

5. How do you feel that the collaboration worked between you, the coach and the person or persons who were responsible for your rehabilitation?

6. Can you give examples of something that you thought worked well regarding the help you received in connection with being injured?

7. Can you give examples of something that you think could be improved regarding the help you received in connection with your injury?

8. How did you experience/think it was to be injured and have difficulty participating or not being able to participate in training/competition?

9. How did you feel during the injury period?

10. Can you tell us about what the decision on returning to sports after injury looked like?

11. Can you tell us about how you felt when you were going to return to sports again? Was there anything that you found difficult, and if so, what?

12. Is there anything else you want to add that you think is important about this area and that we haven't talked about?
